# Behind the Unilateral Rhinorrhea: Delayed Pediatric Intranasal Foreign Bodies Presenting as Chronic Sinonasal Disease and Severe Complications

**DOI:** 10.3390/pediatric18040112

**Published:** 2026-08-11

**Authors:** Constantinos Papadopoulos, Konstantina Dinaki, Ioanna Gravalidou, Rafail Ioannidis

**Affiliations:** 1 Department of ENT Surgery, General Hospital of Drama, 66100 Drama, Greece; constantine.mbg@gmail.com; 2 Department of ENT Surgery, General Hospital of Mytiline ‘Vostaneio’, 81132 Lesvos, Greece; konnidin@windowslive.com; 3 Department of General Surgery, Landesspital Liechtenstein, 9490 Vaduz, Liechtenstein; ioanna_0908@hotmail.com; 4 Department of Anesthesiology and Pain Medicine, General Hospital of Drama, 66100 Drama, Greece

**Keywords:** intranasal foreign body, pediatric otolaryngology, unilateral rhinorrhea, delayed diagnosis, orbital abscess, endoscopic sinus surgery

## Abstract

Background and Clinical Significance: Intranasal foreign bodies are common pediatric otolaryngologic emergencies and are usually diagnosed and removed without difficulty. However, delayed or occult retention may mimic chronic unilateral sinonasal disease and occasionally result in severe inflammatory or infectious complications. This study presents three illustrative pediatric cases of delayed intranasal foreign bodies supported by a seven-year institutional experience. Case Presentation: A retrospective review was conducted of all pediatric patients (≤16 years) presenting with intranasal foreign bodies at a secondary referral hospital between January 2019 and May 2026. Demographic characteristics, clinical presentation, management, and outcomes were reviewed to provide institutional context. Among 82 identified patients, three children with delayed diagnosis and severe complications were selected for detailed presentation because they represented distinct clinical manifestations of prolonged foreign body retention. These included chronic unilateral rhinosinusitis caused by a retained peanut fragment, a medial orbital subperiosteal abscess secondary to a retained plastic nasal piercing component, and a foreign-body granuloma associated with retained nasal packing material. The remaining patients underwent uncomplicated removal, predominantly in the emergency department. Conclusions: The presented cases illustrate the diverse spectrum of complications that may occur following delayed intranasal foreign body retention in children. Persistent unilateral foul-smelling rhinorrhea, nasal obstruction, recurrent unilateral epistaxis, or refractory unilateral sinonasal symptoms should prompt careful evaluation for a retained foreign body. By combining detailed case descriptions with institutional experience, this report highlights the importance of maintaining a high index of suspicion and timely endoscopic management in children with persistent unilateral sinonasal symptoms.

## 1. Introduction

Intranasal foreign bodies are among the most common pediatric otolaryngologic emergencies; however, delayed or occult cases may present an important diagnostic and therapeutic challenge. While nasal foreign bodies are commonly encountered in children because of their exploratory behavior and tendency to insert objects into body orifices, migration or direct placement of foreign materials into the paranasal sinuses occurs infrequently [1,2,3]. Such cases are usually associated with facial trauma, penetrating injuries, dental interventions, previous surgical procedures or accidental insertion of objects [2,4]. However, delayed or occult foreign body retention may present significant diagnostic challenges, particularly when symptoms mimic chronic inflammatory sinonasal disease. Delayed diagnosis may lead to substantial morbidity.

The clinical presentation of retained intranasal foreign bodies in children is often variable and nonspecific. Symptoms depend on several factors, including the size, location, composition and duration of retention of the foreign body. Some patients may remain asymptomatic for extended periods, with the lesion discovered incidentally during radiologic investigations performed for unrelated reasons. However, symptomatic children may present with unilateral nasal obstruction, purulent rhinorrhea, facial pain or swelling, recurrent sinusitis, epistaxis, headache, foul-smelling nasal discharge or fever [1,3,5]. Chronic inflammatory reactions surrounding the retained material may further complicate the clinical picture and mimic other sinonasal disorders, thereby increasing the likelihood of delayed diagnosis. In younger children, the inability to provide a reliable history may make identification even more difficult [6].

Radiologic imaging plays a fundamental role in the diagnosis and management of complicated intranasal foreign bodies. Computed tomography (CT) is considered the imaging modality of choice because it allows accurate localization of the foreign body, assessment of adjacent anatomical structures and evaluation of associated inflammatory or infectious complications. Metallic foreign bodies are generally easy to identify radiologically, whereas organic materials such as wood or plant matter may be more difficult to detect and can sometimes mimic air or soft tissue densities [4,7]. Early and accurate diagnosis is essential to avoid long-term complications and to guide appropriate surgical planning.

Even in asymptomatic patients, removal of intranasal foreign bodies is generally recommended. Retained foreign material may disrupt normal mucociliary clearance and promote persistent mucosal irritation, ultimately leading to chronic rhinosinusitis. Additional complications reported in the literature include mucosal cyst formation, foreign body granuloma, osteomyelitis, fungal colonization, persistent oroantral fistula and chronic facial pain [1,2,4]. In severe or neglected cases, infection may spread to neighboring orbital or intracranial structures, particularly in pediatric patients whose anatomical barriers are thinner and still developing. Therefore, prompt management is considered essential to minimize morbidity and prevent potentially serious sequelae.

The development of endoscopic sinus surgery has significantly improved the management of intranasal foreign bodies in children. Endoscopic techniques allow excellent visualization, precise localization and minimally invasive removal with reduced postoperative morbidity compared with traditional external approaches [6,8]. Nevertheless, because these cases remain uncommon in the pediatric population, the current literature is largely limited to isolated case reports and small case series. Consequently, additional reports are valuable for improving understanding of the etiology, clinical presentation, diagnostic evaluation, treatment options and outcomes associated with pediatric intranasal foreign bodies.

Although uncomplicated pediatric intranasal foreign bodies are frequently reported, severe complications resulting from delayed diagnosis remain uncommon and are described mainly in isolated case reports and small case series.

The present study presents three illustrative pediatric cases of delayed intranasal foreign bodies associated with severe complications, including chronic unilateral rhinosinusitis, orbital subperiosteal abscess, and foreign-body granuloma. These cases are supported by a retrospective review of all pediatric intranasal foreign bodies managed at our institution over a seven-year period, providing epidemiological context and emphasizing the rarity of severe complications. By combining detailed case descriptions with institutional experience, we aim to highlight the diagnostic challenges posed by delayed intranasal foreign bodies and reinforce the importance of maintaining a high index of suspicion in children presenting with persistent unilateral sinonasal symptoms.

## 2. Institutional Experience and Case Selections

A retrospective chart review with presentation of three illustrative complicated cases was performed at the Department of Otolaryngology of the General Hospital of Drama, Greece. Pediatric patients up to 16 years of age presenting to the emergency department between 1 January 2019 and 30 May 2026 with ICD-10 diagnosis code T17.1 (foreign body in nostril) were included in the study. Electronic medical records were reviewed retrospectively. Demographic data, age distribution, clinical manifestations, type of foreign body, management strategy, need for surgical intervention, hospitalization and postoperative outcomes were recorded.

Patients were categorized into four age groups: 8–12 months, 1–3 years, 3–12 years, 12–16 years. Special attention was given to patients presenting with delayed diagnosis, chronic inflammatory manifestations, recurrent symptoms, granulomatous tissue formation or complications requiring surgical intervention under general anesthesia.

Descriptive statistics were used to summarize demographic and clinical characteristics. Categorical variables are presented as frequencies and percentages. Owing to the small number of complicated cases, inferential statistical analyses were not performed. Statistical evaluation was therefore limited to descriptive analysis.

Between 1 January 2019 and 30 May 2026, a total of 82 pediatric patients presented to the emergency department with intranasal foreign bodies (Table 1).

Age distribution demonstrated a predominance of preschool and school-age children (Table 2):

- 8 patients were between 8 and 12 months of age (9.8%);
- 17 patients were between 1 and 3 years (20.7%);
- 50 patients were between 3 and 12 years (61%);
- 7 patients were between 12 and 16 years (8.5%).

Male patients accounted for 34 cases, while female patients represented 48 cases.

The majority of foreign bodies consisted of small plastic toy fragments, predominantly LEGO-type components (Table 3). Most cases were identified and managed immediately in the emergency department with uncomplicated extraction under direct visualization. Five patients had a documented diagnosis of Down syndrome. No statistical analysis regarding developmental conditions was performed because of the limited sample size.

**Table 1:** Demographic and Clinical Characteristics of Pediatric Patients with Intranasal Foreign Bodies.

Variable	Number of Patients (*n* = 82)	Percentage (%)
Male	34	41.5
Female	48	58.5
Total	82	100
Managed in Emergency Department	79	96.3
Required surgery under general anesthesia	3	3.7
Down syndrome	5	6.1
Delayed diagnosis	3	3.7
Orbital complications	1	1.2
Granulomatous inflammatory reaction	3	3.7

**Table 2:** Age Distribution of Pediatric Patients.

Age Group	Number of Patients	Percentage (%)
8–12 months	8	9.8
1–3 years	17	20.7
3–12 years	50	61.0
12–16 years	7	8.5
Total	82	100

**Table 3:** Types of Intranasal Foreign Bodies and Management.

Foreign Body Type	Number	Initial Management	Surgery Required
Plastic toy fragments	Majority	ED removal	None
Organic material (peanut)	1	Failed conservative treatment	Yes
Decorative plastic component	1	Delayed diagnosis	Yes
Retained nasal packing	1	Delayed diagnosis	Yes

Among the 82 pediatric patients identified during the study period, three patients developed severe complications requiring surgical management under general anesthesia (Table 4). Because these cases represented distinct clinical manifestations of delayed intranasal foreign body retention—including chronic unilateral rhinosinusitis, orbital subperiosteal abscess, and foreign-body granuloma—they were selected for detailed presentation as illustrative cases. The remaining cohort is presented to provide institutional and epidemiological context regarding the rarity of these complications.

## 3. Detailed Case Presentations

### 3.1. Case 1

A 6-year-old boy was referred to our department with a four-month history of persistent left-sided nasal obstruction, foul-smelling purulent rhinorrhea, intermittent fever and progressive nasal congestion. During history taking, the patient’s mother recalled a possible episode of foreign body insertion during a birthday party approximately four months earlier, when the child had been playing while eating peanuts. During this period, the child had received four separate courses of oral antibiotics prescribed for presumed recurrent upper respiratory tract infection and chronic rhinosinusitis, with only temporary symptomatic improvement.

**Figure 1:**
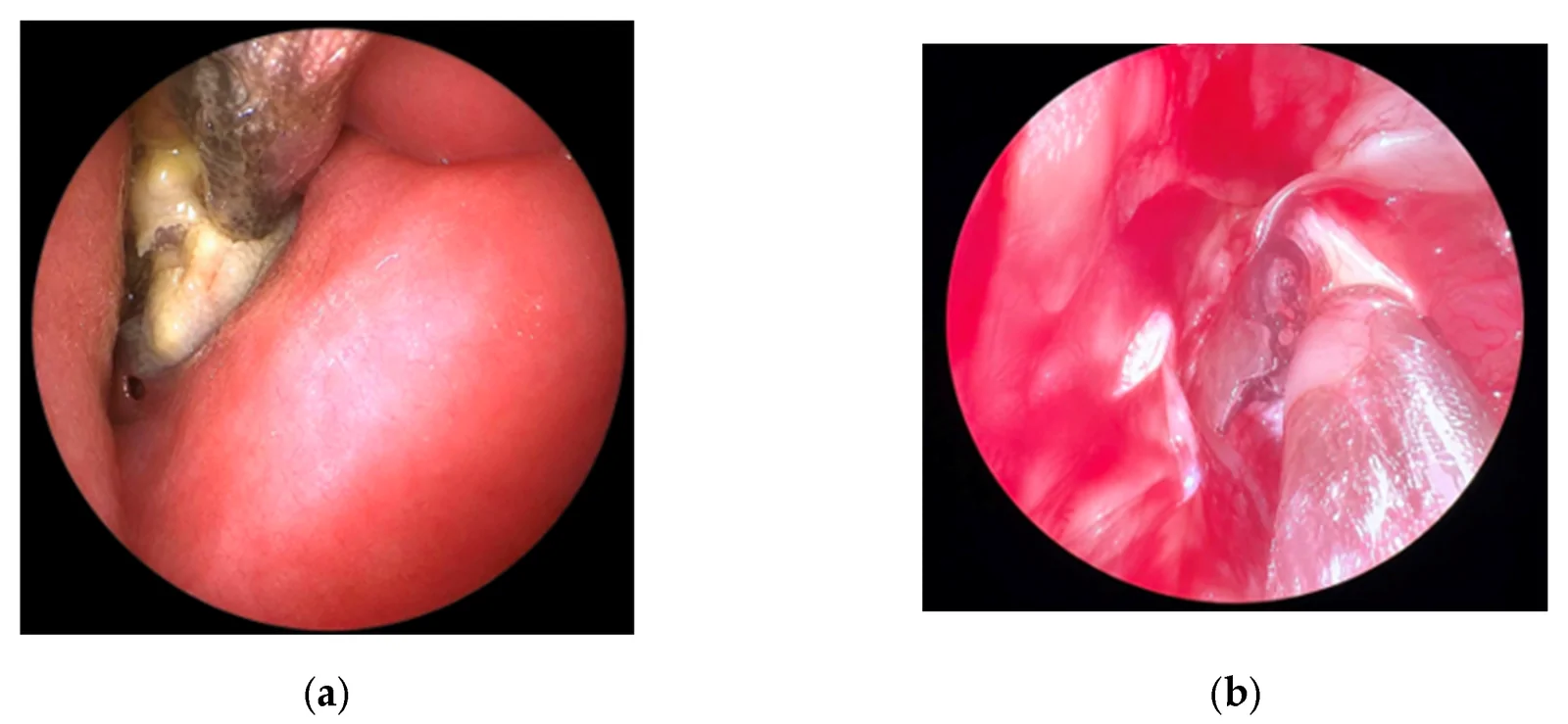
(**a**) Endoscopic view of granulomatous inflammatory tissue and purulent secretions surrounding a retained peanut fragment within the left nasal cavity, (**b**) with intraoperative image during endoscopic ethmoidectomy.

Clinical examination demonstrated unilateral mucopurulent secretions with intense fetid odor originating from the left nasal cavity. Flexible nasal endoscopy revealed extensive purulent secretions and prominent granulomatous inflammatory tissue surrounding an impacted foreign body deeply lodged between the inferior turbinate and the nasal septum. CT demonstrated unilateral inflammatory opacification involving the left nasal cavity and ipsilateral maxillary sinus, consistent with secondary inflammatory rhinosinusitis. (Figure 1).

Under general anesthesia, endoscopic endonasal removal was performed. A retained peanut fragment was identified surrounded by abundant purulent secretions and granulomatous inflammatory tissue. Following careful extraction and endoscopic debridement, rapid clinical improvement was observed with complete resolution of symptoms during follow-up. The patient remained asymptomatic with no evidence of recurrence after 12 months of follow-up.

### 3.2. Case 2

A 13-year-old girl presented to the emergency department with high fever, severe left periorbital edema, facial pain and progressive orbital swelling. According to the parents, intermittent unilateral nasal obstruction and purulent rhinorrhea had been present for several months before acute clinical deterioration.

**Figure 2:**
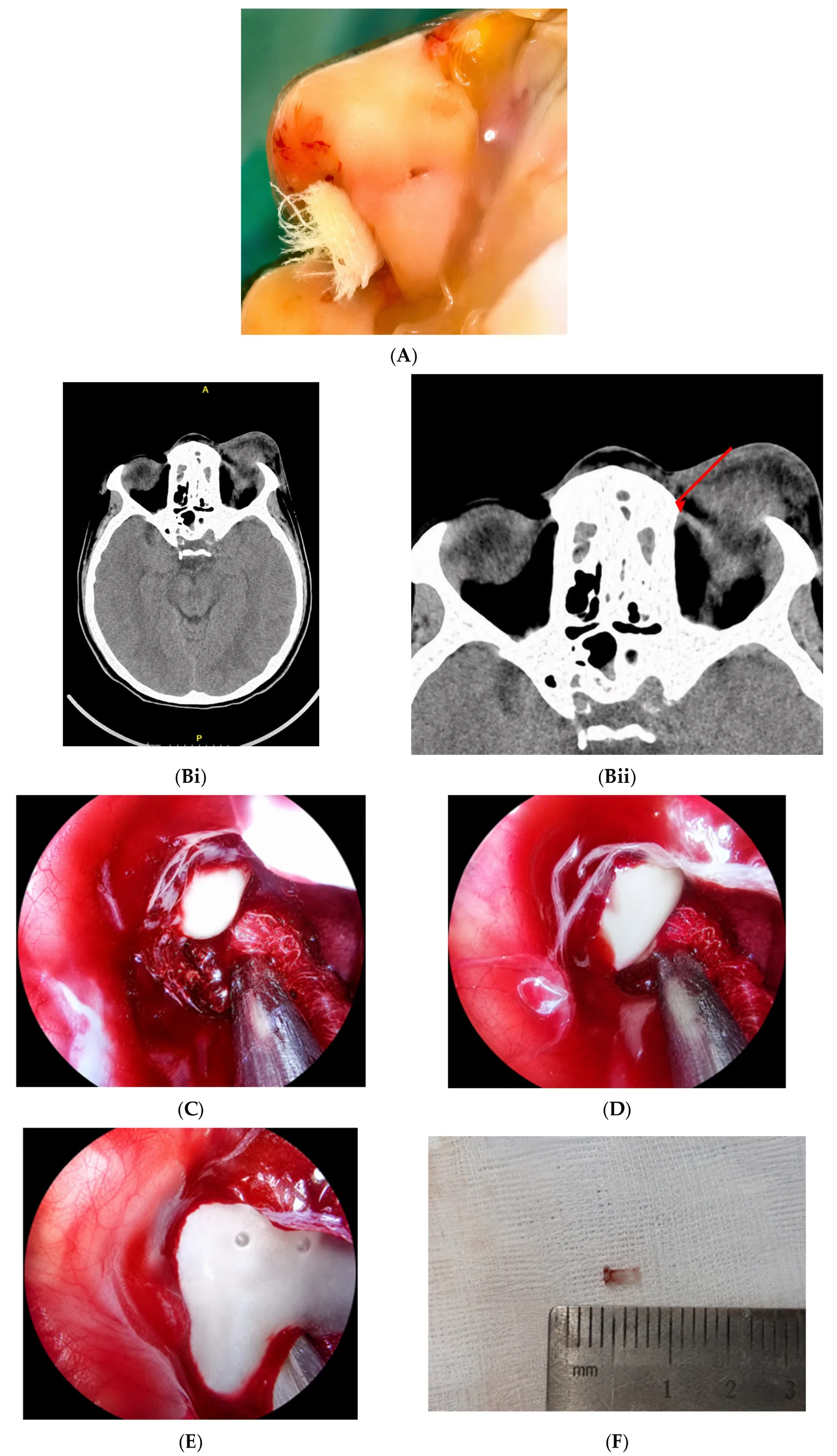
Orbital complication secondary to retained intranasal foreign body. (**A**) External nasal piercing site. (**Bi**) Axial CT showing left ethmoiditis and medial orbital subperiosteal abscess, (**Bii**) Close-up of the (**Bi**) Axial CT showing a more focused view of the subperiosteal abscess (red arrow). (**C**–**E**) Sequential endoscopic views during drainage and debridement. (**F**) Removed plastic locking component.

Ophthalmologic examination revealed painful restriction of ocular motility with preserved visual acuity (10/10). Urgent CT imaging demonstrated extensive ipsilateral ethmoidal sinusitis associated with medial orbital subperiosteal abscess formation. According to the Chandler classification of orbital complications of rhinosinusitis, the lesion was classified as Chandler stage III (subperiosteal abscess). No radiologic evidence of intracranial abscess formation was identified (Figure 2).

The patient underwent emergency combined external and endoscopic surgical treatment. Because the abscess was located superiorly and medially with favorable access through the upper eyelid crease, external drainage of the orbital abscess was performed through an upper eyelid incision, while simultaneous endoscopic sinus surgery included removal of extensive granulomatous inflammatory tissue obstructing the middle meatus, anterior and posterior ethmoidectomy and evacuation of abundant purulent secretions. Further exploration revealed a retained plastic locking component from a decorative nasal piercing deeply embedded within the middle meatal region, acting as the primary nidus for chronic unilateral infection and subsequent orbital complication. Given the severity of the patient’s condition and the absence of neurosurgical support at our institution, the patient was transferred upon completion of the procedure to a quaternary referral center for further treatment and inpatient care.

Postoperatively, dramatic reduction of orbital edema was observed within the first 24 h. Serial imaging demonstrated progressive resolution of inflammatory findings. At 12-month follow-up, ocular motility and visual acuity remained normal, without recurrence of orbital or sinonasal disease.

### 3.3. Case 3

A 14-year-old adolescent boy presented with a six-month history of progressive unilateral nasal obstruction and recurrent ipsilateral epistaxis. The patient reported a previous episode of severe anterior epistaxis managed elsewhere with anterior nasal packing approximately six months before presentation. Clinical examination revealed no systemic inflammatory signs and laboratory investigation demonstrated normal inflammatory markers. Nasal endoscopy identified a unilateral polypoid mass occupying the middle meatal region and significantly altering the normal architecture of the nasal cavity. The lesion demonstrated recurrent contact bleeding during examination. CT revealed a localized soft tissue lesion within the affected nasal cavity without evidence of bony destruction.

**Figure 3:**
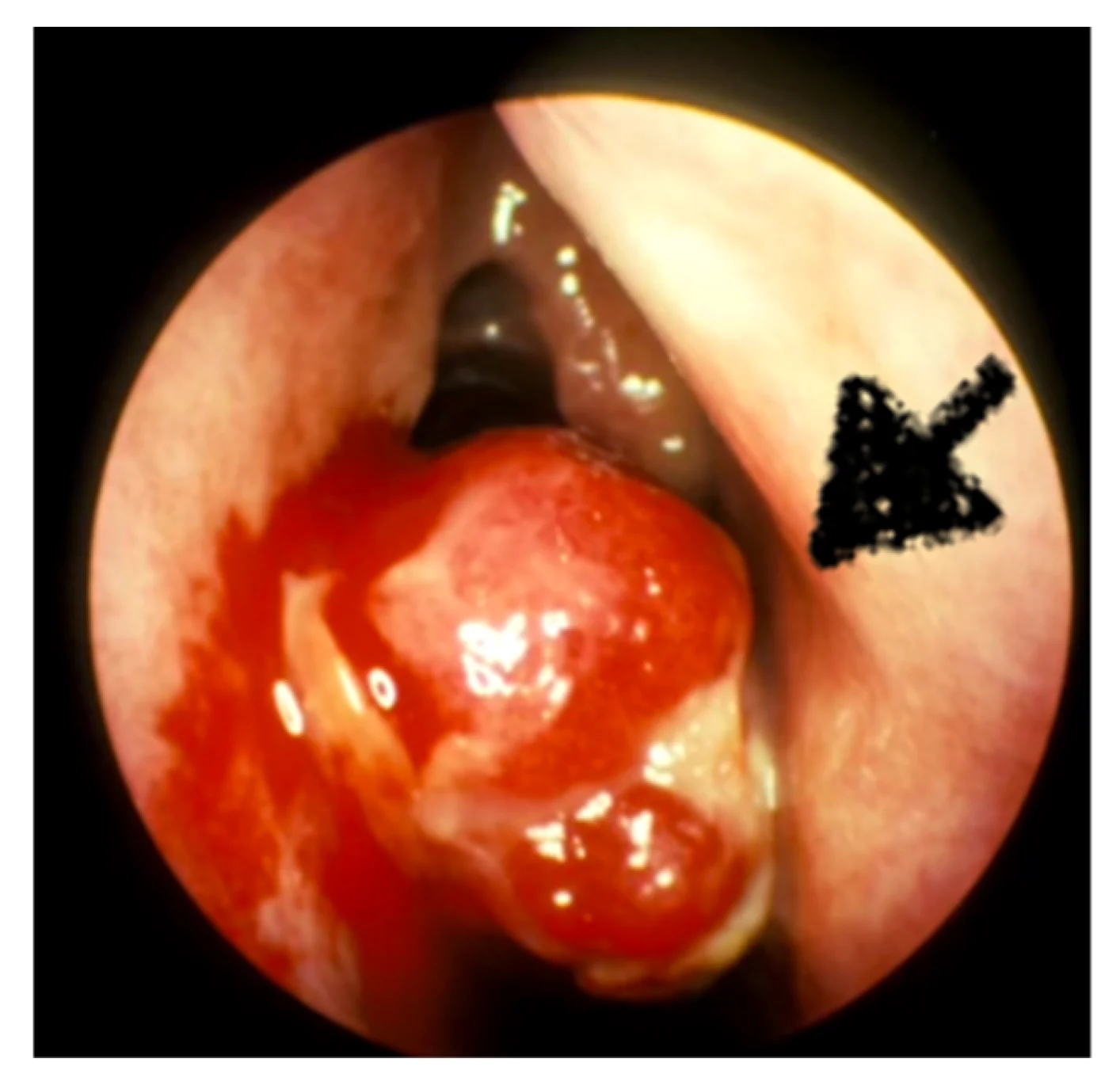
Endoscopic intraoperative image demonstrating granulomatous tissue surrounding retained nasal packing material within the middle meatus, associated with recurrent epistaxis and unilateral nasal obstruction.

The patient underwent endoscopic surgical exploration under general anesthesia. Careful endoscopic dissection of the lesion revealed retained nasal packing material deeply embedded within extensive granulomatous inflammatory tissue originating from the middle meatus. Complete endoscopic removal of both the inflammatory mass and retained packing material was achieved while preserving surrounding sinonasal mucosa. Histopathologic examination confirmed chronic granulomatous inflammatory tissue associated with foreign body reaction surrounding retained packing material (Figure 3).

The postoperative course was uneventful, with complete resolution of nasal obstruction and recurrent epistaxis during follow-up. No recurrence was observed during 12 months of follow-up.

## 4. Discussion

Intranasal foreign bodies account for a substantial proportion of pediatric otolaryngologic emergency visits, particularly among preschool-aged children. Previous studies have consistently demonstrated peak incidence between 2 and 8 years of age, correlating with increased exploratory behavior and exposure to small household or toy-related objects [1,2,3,4,5]. Our findings similarly demonstrated the highest incidence within the 3–12-year age group, accounting for 61% of all presentations. Plastic toy fragments represented the most common foreign bodies in our cohort, reflecting contemporary patterns of pediatric exposure to small detachable toy components. This observation is in agreement with recent reports indicating a shift from predominantly organic foreign bodies toward plastic toy-related materials, likely reflecting changes in children’s play environments and consumer products [2,3,5]. Although our retrospective cohort primarily provides epidemiological context rather than analytical data, these findings confirm that uncomplicated intranasal foreign bodies are common pediatric emergencies, whereas severe complications requiring operative intervention remain distinctly uncommon.

Consistent with previous reports, most patients in our cohort were successfully managed in the emergency department, with only three children (3.7%) requiring surgical intervention under general anesthesia because of delayed diagnosis and prolonged foreign body retention. While the absolute number of complicated cases was small, these patients accounted for the entire spectrum of severe morbidity observed during the study period, including chronic unilateral rhinosinusitis, orbital subperiosteal abscess, and foreign-body granuloma. Rather than representing common clinical presentations, these cases illustrate the uncommon but clinically important consequences that may arise when intranasal foreign bodies remain unrecognized for prolonged periods [1,4,8]. Similar observations have been reported in previous pediatric series, where delayed diagnosis, repeated empirical antibiotic treatment, and persistent unilateral symptoms were consistently associated with an increased likelihood of inflammatory and infectious complications. Accordingly, the educational value of the present report lies not in the frequency of these complications but in demonstrating the diverse clinical manifestations that should prompt clinicians to suspect an occult retained foreign body.

One of the most important observations emerging from our series is the remarkable ability of retained intranasal foreign bodies to mimic chronic unilateral rhinosinusitis. In both Case 1 and Case 2, patients experienced prolonged periods of unilateral sinonasal symptoms before the underlying etiology was identified. During this time, management focused primarily on presumed inflammatory or infectious sinonasal disease rather than on the possibility of a retained foreign body. Similar diagnostic delays have been reported in previous studies, where chronic unilateral rhinorrhea, nasal obstruction, recurrent sinus infections and persistent foul-smelling discharge frequently led to repeated empirical antibiotic treatment before endoscopic examination established the correct diagnosis [1,4,8]. The present findings reinforce the importance of considering retained foreign bodies in the differential diagnosis of any child presenting with persistent unilateral sinonasal symptoms, particularly when symptoms fail to respond to conventional medical therapy.

Several authors have emphasized that unilateral foul-smelling rhinorrhea should be considered highly suggestive of a retained intranasal foreign body until proven otherwise [1,4]. Any child presenting with persistent unilateral foul-smelling rhinorrhea, unilateral nasal obstruction or recurrent unilateral epistaxis should undergo careful nasal endoscopic examination to exclude a retained foreign body before repeated empirical antibiotic treatment is prescribed. Our findings strongly support these recommendations, as all three complicated cases shared a common feature of prolonged unilateral symptoms preceding definitive diagnosis.

The first illustrative case exemplifies the characteristic inflammatory response associated with retained organic foreign bodies. Organic materials such as peanuts, beans, seeds, and other plant-derived objects absorb moisture, expand within the nasal cavity, and rapidly induce mucosal edema, bacterial colonization, and intense inflammatory reactions [1,3,4]. Consequently, they are considerably more likely than inert plastic materials to produce chronic purulent rhinorrhea, secondary bacterial rhinosinusitis, and exuberant granulation tissue. Previous case reports describing retained peanut fragments have documented remarkably similar clinical courses, with repeated empirical antibiotic therapy administered before endoscopic examination ultimately revealed the underlying foreign body. Our patient followed a comparable pattern, experiencing four months of persistent unilateral symptoms despite multiple antibiotic courses before endoscopic removal established the diagnosis.

Beyond reproducing findings already described in the literature, the first case reinforces an important practical message for everyday clinical practice. Repeated medical treatment alone cannot eliminate the underlying source of chronic inflammation when a retained foreign body remains in situ. Instead, definitive resolution depends upon timely recognition and complete removal of the foreign material. In our patient, endoscopic extraction of the retained peanut fragment resulted in rapid clinical improvement and complete resolution of symptoms without recurrence during follow-up, confirming previous observations that surgical removal rather than prolonged antimicrobial therapy represents the cornerstone of treatment in delayed presentations involving organic foreign bodies [9,10].

Orbital complications secondary to retained sinonasal foreign bodies are exceptionally uncommon but potentially life-threatening. Infection may spread from the ethmoid sinus through the thin lamina papyracea into the orbit, resulting in orbital cellulitis or subperiosteal abscess formation. Further extension toward the anterior cranial fossa may lead to cerebritis, meningitis, epidural abscess or intracranial abscess formation [9,10]. According to the Chandler classification, our patient presented with a stage III orbital complication characterized by a medial subperiosteal abscess. This classification remains the most widely accepted system for guiding the assessment and management of orbital complications arising from pediatric rhinosinusitis, with higher stages associated with an increased risk of visual impairment and intracranial extension. Contemporary evidence further supports the role of contrast-enhanced computed tomography as the imaging modality of choice for defining the extent of disease, identifying orbital collections, and planning surgical intervention [11].

Although orbital complications of pediatric rhinosinusitis are well documented, reports directly linking them to occult retained intranasal foreign bodies remain exceedingly rare. Most published cases involve delayed recognition of organic foreign bodies or neglected nasal foreign bodies that acted as persistent infectious foci before progressing to orbital involvement. Our second case illustrates a particularly unusual mechanism, as the underlying nidus consisted of a retained plastic locking component from a decorative nasal piercing rather than an organic object. Despite the inert nature of the material, prolonged retention resulted in chronic unilateral inflammation, ethmoiditis, and ultimately a medial subperiosteal abscess [7,12]. This observation emphasizes that the severity of complications appears to depend not only on the composition of the foreign body but also on the duration of retention and the resulting chronic inflammatory response. Similar observations have been reported in isolated case reports describing occult foreign bodies presenting after prolonged periods with orbital or severe sinonasal complications, highlighting the importance of maintaining a high index of suspicion even when the foreign body itself would not traditionally be considered highly inflammatory [2,13,14]. The successful outcome of this patient underscores the importance of rapid multidisciplinary intervention. Prompt collaboration among otolaryngologists, ophthalmologists, anesthesiologists, pediatricians, radiologists and neurosurgeons enabled early surgical drainage, eradication of the infectious nidus and prevention of permanent visual or neurological sequelae.

The third illustrative case demonstrates another uncommon manifestation of delayed foreign-body retention. Rather than presenting with overt infection, the patient developed a progressive granulomatous inflammatory reaction surrounding retained nasal packing material, ultimately producing a unilateral polypoid mass associated with recurrent epistaxis and nasal obstruction. Similar foreign-body granulomas have been described following prolonged retention of surgical materials within the sinonasal cavity, where chronic inflammatory stimulation may produce exuberant granulation tissue that clinically and radiologically resembles inflammatory polyps or even sinonasal neoplasms. Consequently, differentiation based solely on clinical examination or imaging may be challenging, and definitive diagnosis frequently requires endoscopic exploration with histopathological confirmation [2,4,8,15].

Our third case closely parallels these previously reported observations while also highlighting an important practical lesson. Careful review of the patient’s medical history revealed a previous episode of severe epistaxis managed with anterior nasal packing several months earlier, providing the critical clue to the diagnosis. Without this information, the combination of recurrent epistaxis, unilateral obstruction, and a localized sinonasal mass could reasonably have raised concern for neoplastic disease. Endoscopic surgery therefore served both diagnostic and therapeutic purposes, allowing complete removal of the retained material while preserving the surrounding sinonasal mucosa. This case further illustrates how delayed foreign-body reactions may masquerade as other unilateral sinonasal disorders and reinforces the importance of considering retained surgical materials in the differential diagnosis of persistent unilateral nasal masses, particularly in patients with a history of previous nasal intervention.

The development of endoscopic sinonasal surgery has significantly improved the management of complicated intranasal and paranasal sinus foreign bodies. Compared with traditional external approaches, endoscopic techniques provide superior visualization, precise localization, minimally invasive extraction, preservation of normal sinonasal anatomy and restoration of physiological drainage pathways. Recent advances in pediatric endoscopic sinus surgery have further improved surgical safety and postoperative outcomes [6,16]. In all surgically managed patients in our series, endoscopic treatment resulted in complete clinical recovery without long-term functional complications, further supporting its role as the preferred treatment modality for complicated pediatric sinonasal foreign bodies.

Although endoscopic examination remains the gold standard for diagnosis, simple bedside examination tools may facilitate earlier recognition of intranasal foreign bodies, particularly in emergency departments or primary care settings where nasal endoscopy is not immediately available. An otoscope, which is readily available in virtually all emergency departments, can be used for initial examination of the nasal cavity and has demonstrated moderate sensitivity (57.8%) and high specificity (85.7%) for detecting nasal foreign bodies. Its routine use in children presenting with persistent unilateral rhinorrhea, nasal obstruction, or foul-smelling nasal discharge may increase early detection and reduce delays in diagnosis, thereby facilitating timely referral for definitive endoscopic evaluation when indicated [17].

Beyond the individual clinical scenarios described, the present cases collectively reinforce an important practical message. Although severe complications of intranasal foreign bodies are uncommon, they almost invariably occur in the setting of delayed recognition and prolonged retention. Our institutional experience demonstrates that uncomplicated foreign bodies are usually diagnosed and removed during the initial emergency department visit, whereas the three illustrative cases shared a prolonged history of persistent unilateral symptoms before definitive diagnosis. Rather than representing common presentations, these cases highlight clinical situations in which failure to consider a retained foreign body may result in progressive inflammation, chronic infection, granulomatous reaction, or extension beyond the sinonasal cavity. Maintaining a high index of suspicion is therefore essential, particularly in children with persistent unilateral symptoms that fail to respond to conventional medical treatment.

The present study has several limitations that should be acknowledged. First, its retrospective, single-center design inherently limits the generalizability of the findings. Second, because only three patients developed severe complications, no meaningful statistical analysis of risk factors associated with delayed diagnosis or disease progression could be performed. Furthermore, the institutional cohort was included primarily to provide epidemiological context regarding the rarity of complicated presentations rather than to establish associations between patient characteristics and clinical outcomes. Consequently, the observations presented should be interpreted as descriptive clinical evidence illustrating uncommon but important manifestations of delayed intranasal foreign body retention rather than as evidence of causal relationships.

Nevertheless, the principal strength of this study lies in the detailed presentation of three distinct and well-documented complications arising from delayed diagnosis, each representing a different clinical pathway through which retained intranasal foreign bodies may present. When considered alongside the broader institutional experience, these cases expand the existing literature by illustrating the diverse spectrum of delayed complications—from chronic unilateral rhinosinusitis to orbital subperiosteal abscess and foreign-body granuloma—and emphasize the importance of early recognition, thorough nasal examination, and timely endoscopic management in children presenting with persistent unilateral sinonasal symptoms.

## 5. Conclusions

The present case series, supported by a seven-year institutional experience, highlights the diverse clinical manifestations of delayed intranasal foreign body retention in children. Although severe complications were uncommon within our cohort, the three illustrative cases demonstrate that prolonged retention may present as chronic unilateral rhinosinusitis, orbital subperiosteal abscess, or foreign-body granuloma, often mimicking more common sinonasal disorders and contributing to delayed diagnosis. Persistent unilateral foul-smelling rhinorrhea, nasal obstruction, recurrent unilateral epistaxis, or refractory unilateral sinonasal symptoms should prompt careful examination of the nasal cavity and consideration of a retained foreign body, particularly in young children or when symptoms fail to respond to appropriate medical therapy. Early recognition, appropriate imaging when indicated, and timely endoscopic management were associated with excellent outcomes in the present cases.

By combining detailed case descriptions with institutional experience, this report expands the existing literature on uncommon complications of pediatric intranasal foreign bodies and reinforces the importance of maintaining a high index of suspicion for this readily treatable condition in children presenting with persistent unilateral sinonasal symptoms.

## Figures and Tables

**Table 4:** Summary of Complicated Cases.

Case	Age/Sex	Foreign Body	Duration of Symptoms	Main Presentation	Complication	Treatment	Outcome
1	6/M	Peanut fragment	4 months	Unilateral foul-smelling rhinorrhea and obstruction	Chronic unilateral rhinosinusitis	Endoscopic removal	Complete recovery
2	13/F	Plastic nasal piercing locking component	Several months	Periorbital edema, fever, orbital swelling	Medial orbital subperiosteal abscess	External drainage + ESS	Complete recovery
3	14/M	Retained nasal packing	6 months	Recurrent epistaxis and obstruction	Foreign body granuloma	Endoscopic excision	Complete recovery

## Data Availability

The original contributions presented in this study are included in the article. Further inquiries can be directed to the corresponding author.

## Abbreviations

The following abbreviations are used in this manuscript:

CT	Computed Tomography

## References

[1] Baranowski K., Al Aaraj M.S., Sinha V. (2023). Nasal Foreign Body. StatPearls [Internet].

[2] Paladin I., Mizdrak I., Gabelica M., Golec Parčina N., Mimica I., Batinović F. (2024). Foreign Bodies in Pediatric Otorhinolaryngology: A Review. Pediatr. Rep..

[3] Cetinkaya E.A., Arslan İ.B., Cukurova İ. (2015). Nasal foreign bodies in children: Types, locations, complications and removal. Int. J. Pediatr. Otorhinolaryngol..

[4] Patil P.M., Anand R. (2011). Nasal Foreign Bodies: A Review of Management Strategies and a Clinical Scenario Presentation. Craniomaxillofac. Trauma Reconstr..

[5] Hira İ., Tofar M., Bayram A., Yaşar M., Mutlu C., Özcan İ. (2019). Childhood Nasal Foreign Bodies: Analysis of 1724 Cases. Turk. Arch. Otorhinolaryngol..

[6] Jungbauer W.N., Shih M., Nguyen S.A., Clemmens C.S. (2022). Comparison of pediatric nasal foreign body removal by care setting: A systematic review and meta-analysis. Int. J. Pediatr. Otorhinolaryngol..

[7] Asiri M., Al-Khulban M.S., Al-Sayed G. (2023). Foreign Body in the Nasal Cavity: A Case Report. Cureus.

[8] Yan S., Chen G., Zeng N., Gao C. (2024). Characteristics and treatment of pediatric nasal foreign bodies with button batteries-A retrospective analysis of 176 cases. PLoS ONE.

[9] Bahranifad H., Zandifar Z., Zaheri P.M., Wallin L., Karimi Akhormeh A., Parsa N. (2022). Prolonged Undiagnosed Nasal Foreign Body Case Report. Indian J. Otolaryngol. Head Neck Surg..

[10] Jalaladdin M., Alaidarous S.T., Althqafi A., Babkour A.A. (2025). Delayed Diagnosis of a Nasal Foreign Body (Battery) Retained for 13 Years. Ear Nose Throat J..

[11] Torretta S., Guastella C., Marchisio P., Marom T., Bosis S., Ibba T., Drago L., Pignataro L. (2019). Sinonasal-Related Orbital Infections in Children: A Clinical and Therapeutic Overview. J. Clin. Med..

[12] Heilig Y., Yafit D., Schneider S., Kordeluk S., Ziv O. (2024). Bilateral orbital abscess secondary to pediatric acute rhinosinusitis: A case report and literature review. J. AAPOS.

[13] Sciarretta V., Demattè M., Farneti P., Fornaciari M., Corsini I., Piccin O., Saggese D., Fernandez I.J. (2017). Management of orbital cellulitis and subperiosteal orbital abscess in pediatric patients: A ten-year review. Int. J. Pediatr. Otorhinolaryngol..

[14] Din-Lovinescu C., Mir G., Blanco C., Zhao K., Mazzoni T., Fried A., El Khashab M., Lin G. (2020). Intracranial complications of pediatric rhinosinusitis: Identifying risk factors and interventions affecting length of hospitalization. Int. J. Pediatr. Otorhinolaryngol..

[15] Talugula S., Callahan N., Lee V.S. (2025). A Rare Cause of Chronic Rhinosinusitis Secondary to a Retained Pledget: A Case Report. Case Rep. Otolaryngol..

[16] Purrinos J.A., Younis R. (2024). Pediatric endoscopic sinus surgery: Revisited 35 years later. Am. J. Otolaryngol..

[17] Chainansamit S., Chit-Uea-Ophat C., Reechaipichitkul W., Piromchai P. (2021). The Diagnostic Value of Traditional Nasal Examination Tools in an Endoscopic Era. Ear Nose Throat J..

